# Functional heartburn has more in common with functional dyspepsia than with non-erosive reflux disease

**DOI:** 10.1136/gut.2008.175810

**Published:** 2009-05-20

**Authors:** E Savarino, D Pohl, P Zentilin, P Dulbecco, G Sammito, L Sconfienza, S Vigneri, G Camerini, R Tutuian, V Savarino

**Affiliations:** 1 Division of Gastroenterology, Department of Internal Medicine, University of Genoa, Genoa, Italy; 2 Division of Gastroenterology and Hepatology, Department of Internal Medicine, University Hospital Zurich, Zurich, Switzerland; 3 Division of Gastroenterology, Department of Internal Medicine, University of Palermo, Palermo, Italy; 4 Division of Surgery, Department of Internal Medicine, University of Genoa, Genoa, Italy

## Abstract

**Introduction:**

Functional dyspepsia and non-erosive reflux disease (NERD) are prevalent gastrointestinal conditions with accumulating evidence regarding an overlap between the two. Still, patients with NERD represent a very heterogeneous group and limited data on dyspeptic symptoms in various subgroups of NERD are available.

**Aim:**

To evaluate the prevalence of dyspeptic symptoms in patients with NERD subclassified by using 24 h impedance-pH monitoring (MII-pH).

**Methods:**

Patients with typical reflux symptoms and normal endoscopy underwent impedance-pH monitoring off proton pump inhibitor treatment. Oesophageal acid exposure time (AET), type of acid and non-acid reflux episodes, and symptom association probability (SAP) were calculated. A validated dyspepsia questionnaire was used to quantify dyspeptic symptoms prior to reflux monitoring.

**Results:**

Of 200 patients with NERD (105 female; median age, 48 years), 81 (41%) had an abnormal oesophageal AET (NERD pH-POS), 65 (32%) had normal oesophageal AET and positive SAP for acid and/or non-acid reflux (hypersensitive oesophagus), and 54 (27%) had normal oesophageal AET and negative SAP (functional heartburn). Patients with functional heartburn had more frequent (p<0.01) postprandial fullness, bloating, early satiety and nausea compared to patients with NERD pH-POS and hypersensitive oesophagus.

**Conclusion:**

The increased prevalence of dyspeptic symptoms in patients with functional heartburn reinforces the concept that functional gastrointestinal disorders extend beyond the boundaries suggested by the anatomical location of symptoms. This should be regarded as a further argument to test patients with symptoms of gastro-oesophageal reflux disease in order to separate patients with functional heartburn from patients with NERD in whom symptoms are associated with gastro-oesophageal reflux.

Gastro-oesophageal reflux disease (GORD) is one of the most common chronic gastrointestinal diseases in Western countries.^1^,^2^ Recent studies documented that up to 70% of reflux patients have typical reflux symptoms (ie, heartburn and/or regurgitation) in the absence of endoscopically visible oesophageal mucosal injuries, making non-erosive reflux disease (NERD) the more common form of GORD.^3^,^4^ The NERD patient group incorporates subgroups which differ significantly in terms of presentation, pathophysiology and management. Patients experiencing typical reflux symptoms without evidence of oesophagitis on upper endoscopy are classified on the basis of oesophageal pH monitoring results and symptom association analysis as suffering either from NERD, when excessive acid reflux or a positive symptom association with acid reflux is demonstrated, or from functional heartburn (FH), when, in agreement with Rome III criteria, distal oesophageal acid exposure is normal and a negative response to acid suppression is found.^5^,^6^ Recognising that stimuli other than acid can evoke typical reflux symptoms,^7^ our group previously proposed subclassifying patients with typical reflux symptoms and normal upper gastrointestinal endoscopy as follows: (1) NERD pH-POS patients with normal endoscopy and abnormal distal oesophageal acid exposure; (2) hypersensitive oesophagus – patients with normal endoscopy, normal distal oesophageal acid exposure and positive symptom association for either acid or non-acid reflux; and (3) functional heartburn – patients with normal endoscopy, normal distal oesophageal acid exposure and negative symptom association for acid and non-acid reflux.^8^

Patients with GORD, both with erosive oesophagitis and NERD, frequently report dyspeptic symptoms.^9^ Epidemiological studies investigating the prevalence of dyspeptic and oesophageal symptoms have reported a higher prevalence of dyspeptic symptoms in patients with GORD, suggesting that the degree of overlap is greater than could be predicted by chance alone.^10^ Moreover, it was recently demonstrated that patients with functional heartburn and poor response to acid suppressive therapy are more likely to have psychopathology similarly to patients with functional dyspepsia.^11^ Last, but not least, abdominal symptoms appear to be independent predictors of the severity of reflux symptoms in patients with NERD when compared to normal controls.^12^

Given previous reports indicating an inverse relationship between dyspeptic symptoms and the objective criteria for GORD we hypothesised that in patients with functional heartburn dyspeptic symptoms should be more prevalent compared to the rest of NERD patients. To test this hypothesis, we evaluated the prevalence of dyspeptic symptoms in patients with NERD subclassified into three distinct groups by using 24 h MII-pH monitoring.

## Methods

### Study subjects

Between June 2004 and September 2008, patients presenting to the outpatient motility centre at the University of Genoa with predominant typical GORD symptoms (ie, heartburn and regurgitation) lasting for more than 6 months and occurring at least three times weekly, were prospectively enrolled in the study. All subjects who agreed to participate in our investigation underwent careful history taking physical and clinical examination, upper gastrointestinal endoscopy to assess the presence or not of oesophageal mucosal injury, routine biochemistry, and upper abdominal ultrasound. The medical history included information on treatment with acid suppressive medication (in particular proton pump inhibitors (PPIs)) and symptomatic response to PPI therapy. Patients reporting ⩾50% symptom improvement were considered responders to PPI therapy. Patients treated with antisecretory drugs were asked to discontinue acid suppressive therapy at least 30 days before the endoscopic examination. During the washout period, patients were allowed to use an oral antacid or alginate on an as-needed basis for the relief of heartburn. Based on the results of upper endoscopy, patients were then subdivided into three major groups – Barrett’s oesophagus, erosive oesophagitis and NERD – in cases where the typical symptoms of GORD were present, and where visible oesophageal mucosal injury was absent. Patients with Barrett’s oesophagus and erosive oesophagitis were not included in the present study. Within 1–5 days (median 3 days) from the upper endoscopy, patients with NERD underwent ambulatory combined impedance-pH monitoring. Exclusion criteria were: history of thoracic, oesophageal or gastric surgery; primary or secondary severe oesophageal motility disorders (eg, achalasia, scleroderma, diabetes mellitus, autonomic or peripheral neuropathy, myopathy); underlying psychiatric illness; use of non-steroidal anti-inflammatory drugs (NSAIDs) and aspirin; presence of peptic stricture and duodenal or gastric ulcer on upper endoscopy, evidence of erosive oesophagitis at previous (2–5 years) endoscopy, presence of dyspeptic symptoms as major symptoms. In women of childbearing age, pregnancy was excluded by urine analysis. During upper gastrointestinal endoscopy, biopsies were taken from the antrum and the corpus for assessing the presence of *Helicobacter pylori*. Patients were asked to discontinue any medication that would influence oesophageal motor function at least 1 week before administering the questionnaires and performing tests of oesophageal function.

All participants gave written informed consent before entering the study.

### Symptom questionnaire

Before the 24 h pH-impedance study, each patient completed a functional dyspepsia questionnaire as reported and validated previously.^13^ This questionnaire included questions on the presence and intensity (range, 0–3; where 0 = absent, 1 = mild, 2 = moderate, and 3 = severe, interfering with daily activities) of epigastric pain, bloating, postprandial fullness, early satiety, nausea, vomiting, excessive belching and epigastric burning. Also, typical GORD symptoms (ie, heartburn and regurgitation) were evaluated using the same questionnaire (0 = absent, 1 = mild, 2 = moderate, and 3 = severe). A second investigator completed a structured interview with the patient including a careful medical history (including height and weight), current medication, tobacco use and alcohol consumption.

### Oesophageal multichannel intraluminal impedance and pH monitoring

Oesophageal impedance-pH monitoring was performed using an ambulatory multichannel intraluminal impedance and pH (MII-pH) monitoring system (Sleuth; Sandhill Scientific, Highland Ranch, Colorado, USA). The system included a portable data logger and a catheter with one antimony pH electrode and eight impedance electrodes at 2, 4, 6, 8, 10, 14, 16 and 18 cm from the tip of the catheter. Each pair of adjacent electrodes represented an impedance-measuring segment (2 cm length) corresponding to one recording channel. The six impedance and one pH signals were recorded at 50 Hz on a 128 MB CompactFlash (SanDisk, Milpitas, California, USA).

The methodology of probe calibration, catheter placement, patient instruction and performance have been previously described.^8^ On the monitoring day, each subject ate three standard meals of a Mediterranean diet as previously reported.^14^

The data stored on the CompactFlash card were downloaded into a personal computer and analysed using a semiautomated reflux detection algorithm (Autoscan; Sandhill Scientific). Tracings were subsequently reviewed manually by an expert reader (ES) in order to ensure accurate detection and classification of reflux episodes. Meal periods were excluded from the analysis.

#### Definitions of reflux episodes

Simultaneously recorded pH data were used to classify reflux episodes as acid, weakly acidic, or weakly alkaline according to the previously reported criteria:^15^

- *Acid reflux*: impedance-detected reflux episodes with a nadir pH less than 4
- *Weakly acidic reflux*: impedance-detected reflux episodes with a nadir pH between 4 and 7
- *Weakly alkaline* *reflux*: impedance-detected reflux episodes with a nadir pH above 7. For symptom analysis, weakly acidic and weakly alkaline reflux were grouped together as non-acid reflux episodes (nadir pH>4)

#### Gastro-oesophageal reflux parameters

Impedance and pH data were used to determine the number and type of reflux episodes and distal oesophageal acid exposure (reflux time (min) and reflux per cent time) in each patient.

Total 24 h oesophageal acid exposure (%) was defined as the total time when the pH was below 4 divided by the time of monitoring. A total distal oesophageal acid exposure (ie, per cent time at pH <4) of less than 4.2% over 24 h was considered normal.^8^,^14^

#### Symptom–reflux association analysis

In each patient we calculated the symptom association probability (SAP) for typical oesophageal symptoms. In the analysis we separated symptoms associated with acid reflux from those associated with non-acid reflux (including weakly acidic and weakly alkaline reflux as a whole) and symptoms occurring independent of reflux episodes. Separate analysis was performed for each individual symptom if patients recorded different types of symptoms.

The SAP was calculated for acid and non-acid reflux using a custom made Excel macro function (RT). The algorithm counted the number of symptoms preceded by a reflux episode within 2 min (S+R+). The number of symptoms not associated with reflux (S+R−) was calculated by subtracting S+R+ from the total number of symptoms. The study was divided into 2-min segments in order to determine the number reflux containing segments (R+) and reflux-free symptoms (R−). The two other cells of the 2×2 contingency table (S−R− and S−R+) were calculated by performing the corresponding subtractions. A two-sided Fisher exact test was used to calculate the probability (p) that the observed distribution could have been occurred by chance. SAP was calculated as (1−p)×100% and was considered to be positive when ⩾95% was positive.^8^

### Statistical analysis

Continuous variables were compared using ANOVA (with post hoc comparisons between groups) or the Kruskal–Wallis test depending on the normal or abnormal distribution of data (assessed by the Kolmogorov–Smirnov test). The χ^2^ test was used when comparing categorical variables (ie, the presence/absence of *Helicobacter pylori* infection and individual dyspeptic symptoms). Differences were considered statistically significant when p<0.05.

## Results

Two hundred and sixty-six patients with typical symptoms of GORD met the enrolment criteria and entered the study. Based on the results of upper endoscopy, eight patients had Barrett’s oesophagus, 44 had erosive oesophagitis, and 214 had NERD. Patients with Barrett’s oesophagus and erosive oesophagitis were not included in the present study. Of the patients with NERD, 200 (105 females, mean age 48 years, range 18–78) reported at least one type of typical gastro-oesophageal reflux symptom during the monitoring period and were included in the final analysis. Thirty-four patients (17%) never received PPIs (“PPI naive” NERD patients). Combined impedance-pH monitoring was well tolerated by all subjects and no important technical failure occurred. The median total recording time was 23.4 h (range 22.9–23.6 h).

### Acid exposure and symptom association

**Figure 1:**
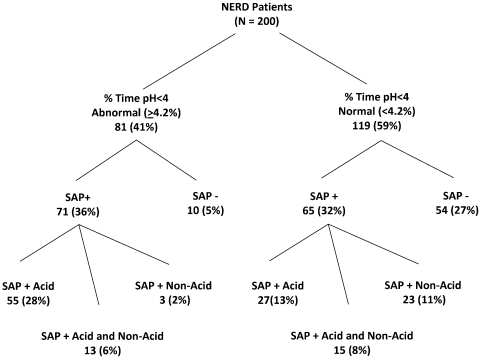
Subclassification of patients with non-erosive reflux disease (NERD), based on distal oesophageal acid exposure and symptom association in NERD pH-POS (abnormal per cent time the pH was <4), hypersensitive oesophagus (normal per cent time pH <4 and positive symptom association probability (SAP), and functional heartburn (normal per cent time the pH was <4, and negative SAP).

Acid exposure time and symptom association probability is shown in fig 1. Patients with NERD were subdivided into four different subgroups according to oesophageal acid exposure and symptom association. We found an abnormal distal oesophageal acid exposure time in 81 (41%) patients, 71 (36%) of whom had a positive SAP (NERD pH-POS/SAP+) and 10 (5%) had no association between symptoms and any type of reflux (NERD pH-POS/SAP−). One hundred and nineteen patients (59%) had a normal distal oesophageal acid exposure (per cent time pH <4 less than 4.2%), 65 (32%) of whom had a positive SAP (NERD pH-NEG/SAP+; hypersensitive oesophagus subgroup). Fifty-four (27%) patients had no association between symptoms and any type of reflux (NERD pH-NEG/SAP−; functional heartburn subgroup).

### Demographic and clinical characteristics

Detailed demographic and clinical characteristics of the various subgroups of patients with NERD are shown in table 1. There were no differences with respect to age, smoking, alcohol, coffee consumption and prevalence of *H pylori* infection (p = NS). The prevalence of hiatal hernia was higher in NERD pH-POS patients with positive SAP and negative SAP compared to those with hypersensitive oesophagus or FH (76.1 vs 80 vs 43.1 vs 42.6; p<0.01). Moreover, patients in the NERD pH-POS subgroups had a higher mean body mass index than patients with hypersensitive oesophagus or FH (26.7 vs 27.3 vs 23.5 vs 22.9; p<0.01). Finally, patients of the functional heartburn subgroup were more frequently women than those in the other two subgroups (40 vs 40.8 vs 53.8% vs 68.5; p<0.01).

**Table 1:** Demographic and clinical characteristics, treatment with proton pump inhibitors (PPIs), and treatment response of patients with non-erosive reflux disease (n = 200)

Demographic or clinical parameter	pH-POS/SAP+	pH-POS/SAP−	pH-NEG/SAP+	pH-NEG/SAP−	p Value
Patients, no	71	10	65	54	
Female patients, no	29	4	35	37	<0.01
Patients with clinically relevant dyspeptic symptoms, no	26	4	24	34	<0.01
Mean age, years (range)	50.7 (20–78)	51.9 (34–76)	46.1 (22–77)	45.8 (18–76)	NS
Mean body mass index (range)	26.7 (18–42)	27.3 (20–38)	23.5 (19–41)	22.9 (16–34)	<0.01
Tobacco use, %	21.1	30.0	23.1	25.9	NS
Alcohol consumption, %	40.8	40.0	33.8	35.2	NS
Coffee consumption, %	80.3	80.0	73.8	77.8	NS
Prevalence of hiatal hernia, %	76.1	80.0	43.1	42.6	<0.01
Prevalence of *Helicobacter pylori* infection, %	8.5	10.0	9.2	9.3	NS
Patients having previously received PPIs, no (%)	46 (65)	6 (60)	62 (95)	52 (96)	<0.01
Positive (⩾50%) symptom response, no (%)	34 (74)	4 (67)	33 (53)	15 (29)	<0.01

NEG, negative; POS, positive; SAP, symptom association probability.

### Typical GORD symptoms in different subgroups of patients with NERD

Patients with NERD had a median heartburn score of 2 (range 1–3) and a median regurgitation score of 2 (range 0–3) recorded in the GORD symptoms questionnaires. Details on the number and percentages of patients on PPIs and the numbers and percentages of patients who responded to PPIs are shown in table 1.

Symptoms reported during the study day were in agreement with the symptom questionnaires. Indeed, 66 (93%) patients with NERD pH-POS/SAP+ (median heartburn score 2), eight (80%) with NERD pH-POS/SAP− (median heartburn score 2), 58 (89%) NERD pH-NEG/SAP+ (median heartburn score 2) and 50 (93%) NERD pH-NEG/SAP− (median heartburn score 2) reported heartburn, while 43 (61%) patients with NERD pH-POS/SAP+ (median regurgitation score 2), five (50%) with NERD pH-POS/SAP− (median regurgitation score 2), 36 (55%) NERD pH-NEG/SAP+ (median regurgitation score 2) and 18 (33%) NERD pH-NEG/SAP− (median regurgitation score 1) reported regurgitation.

**Figure 2:**
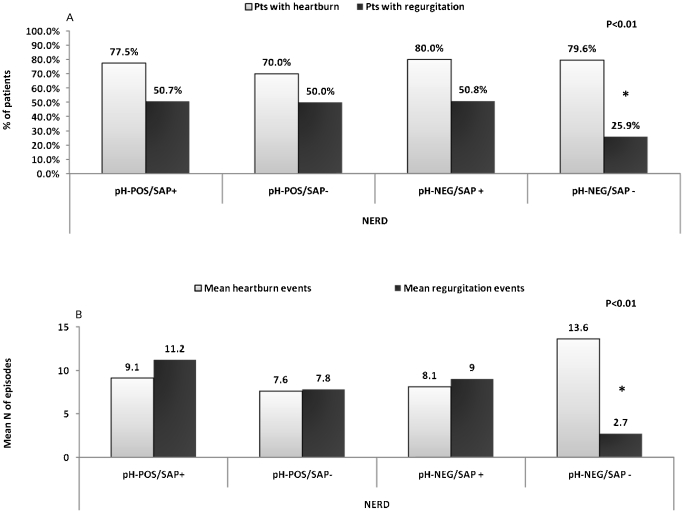
(A) Percentages of patients reporting typical symptoms of gastro-oesphageal reflux disease (GORD) stratified according to the results (n = 200) of 24 h impedance-pH monitoring. (B) Mean numbers of GORD symptoms stratified according to the results (n = 200) of 24 h impedance-pH monitoring. NEG, negative; POS, positive; Pts, patients; SAP, symptom association probability.

The total number of symptoms reported by patients with NERD was 2245 (mean 4.5, range 1–69). Patients reported 1465 heartburn events (mean 10, range 1–62) and 780 regurgitation episodes (mean 9, range 1–69). Data on the number of heartburn and regurgitation events reported by patients in various subgroups of NERD are summarised in fig 2A,B.

### Dyspepsia symptoms in different subgroups of patients with NERD

**Figure 3:**
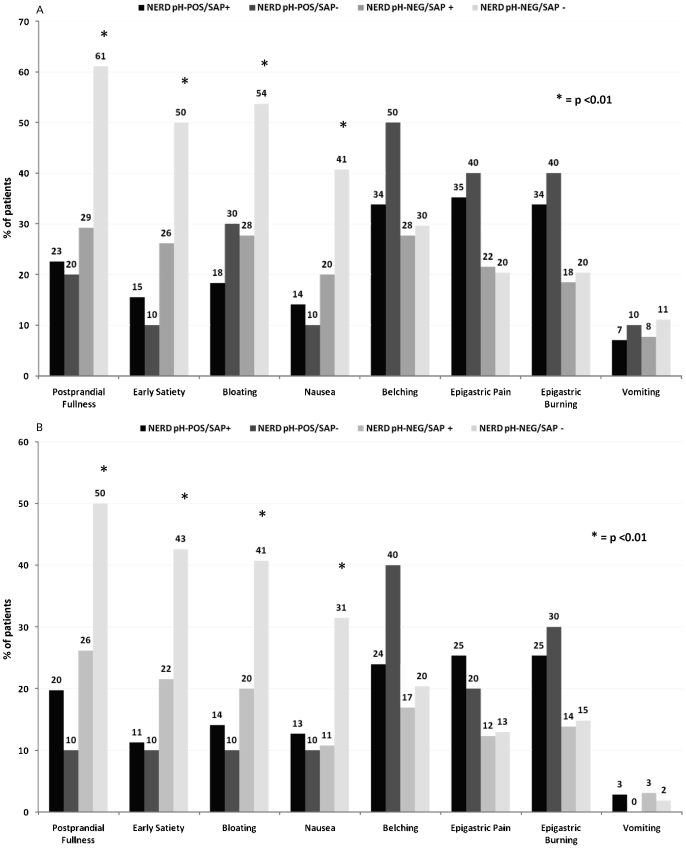
(A) Prevalence of dyspeptic symptoms (score >0) in patients with non-erosive reflux disease (NERD) subclassified using impedance-pH monitoring (n = 200). (B) Prevalence of moderate/severe dyspeptic symptoms (score ⩾2) in patients with NERD subclassified using impedance-pH monitoring (n = 200). NEG, negative; POS, positive; SAP, symptom association probability.

As summarised in fig 3A, in the FH subgroup symptoms such as nausea, postprandial fullness, early satiety and bloating were more frequent compared to NERD pH-POS patients with both positive and negative SAP and those with hypersensitive oesophagus (p<0.01). Epigastric pain and epigastric burning tended to be more frequent in patients of NERD pH-POS with positive SAP and negative SAP subgroups, but statistical significance was not reached (p = NS). Similar results were obtained when considering only moderate/severe dyspeptic symptoms (severity score ⩾2), as shown in fig 3B.

## Discussion

Upper gastrointestinal symptoms are remarkably common in the general population, with the majority of patients having GORD or dyspepsia.^10^,^16^ Periodically updated international criteria, applying standardised questionnaires for symptom collection, have led to the classification of patients into distinct disease categories (currently, Rome III). Patients suffering from symptomatic heartburn and/or regurgitation are clinically classified as GORD – in the absence of oesophageal mucosa abnormalities as NERD – and not dyspepsia. In functional gastrointestinal disorders, however, a certain degree of overlapping symptoms has been reported,^17^,^18^,^19^,^20^ and this degree of overlap appears to be greater than would be predicted by chance alone.

Non-erosive or negative-endoscopy reflux disease (NERD) may account for up to 70% of patients with GORD in the community.^8^,^9^,^10^ NERD is a heterogeneous disorder and incorporates subgroups which differ significantly in terms of presentation, pathophysiology and management. Because of this heterogeneity, previous studies tried to differentiate NERD patients on the basis of acid exposure on 24 h pH studies. However, this differentiation left some difficulties in distinguishing patients with true reflux disease from those with functional disease, reducing the possibility to evaluate the degree of overlap between NERD and functional dyspepsia or functional disorders as a whole. Recently, recognising that stimuli other than acid can evoke typical reflux symptoms,^7^ we have proposed subclassifying patients with NERD by using the combination of conventional pH with oesophageal intraluminal electrical impedance monitoring, in order to distinguish these patients more clearly on the basis of their distal oesophageal acid exposure or positive and/or negative symptom association to acid or non-acid reflux during the MII-pH recording. Applying this technique to the NERD population in current study, we reduced the proportion of patients who would have been previously labelled as presenting with FH as follows: (1) 41% as NERD pH-POS patients; (2) 32% as hypersensitive oesophagus patients; and (3) 27% as functional heartburn patients. These percentages are in agreement with those of a previous study in which we assessed 150 patients with NERD recruited in three Italian centres.^8^

In our study, prospectively collected 24 h ambulatory impedance-pH monitoring data and functional dyspepsia questionnaires in a large group of unselected NERD patients off PPI treatment led us to show that clinically relevant dyspeptic symptoms are present in 44% of the NERD population, in accordance with other investigations.^18^ Moreover, to our knowledge, this is the first study assessing in a large group of patients with NERD, off medication, the distribution of typical GORD and dyspepsia symptoms to evaluate the degree of overlap between functional dyspepsia and the different subgroups of the NERD population identified by means of MII-pH monitoring. In particular, we showed that subclassifying NERD patients into three different groups on the basis of 24 h pH-impedance results, dyspeptic symptoms are more frequently reported in patients with FH (63%) compared to the NERD pH-POS/SAP+ (37%), NERD pH-POS/SAP− (36.6%) and hypersensitive oesophagus (37%) subgroups, suggesting a significantly different degree of overlap of NERD subgroups with functional dyspepsia.

Patients now diagnosed with FH from the NERD collective reported much more frequently dyspeptic symptoms such as postprandial fullness, bloating, early satiety and nausea compared to the NERD pH-POS with positive SAP and negative SAP and hypersensitive oesophagus subgroups. In particular, postprandial fullness and early satiation were the main symptoms more frequently related to the FH subgroup, suggesting a possible association between this disorder and the post prandial distress syndrome, as defined by Rome III criteria.^17^,^21^ The same proportions in symptom prevalence were maintained when considering moderate/severe dyspeptic symptoms, and this further corroborates the fact that dyspeptic symptoms have a different prevalence among the various subgroups of NERD patients we identified by means of MII-pH. Moreover, we found that symptoms such as epigastric pain and epigastric burning were more frequently encountered in NERD pH-POS with positive SAP and negative SAP patients, thus confirming previous controlled studies showing that epigastric pain syndrome is more prevalent in patients with abnormal pH test.^22^ This is in keeping with findings from controlled studies showing that NERD patients with a positive pH-metry are more likely to respond to proton pump inhibitor treatment, similarly to dyspeptic patients with predominant pain.^23^ Finally, the presence of a different distribution of dyspepsia symptoms in our patients confirms the existence of two different clinical dyspepsia patterns as assessed in the last Rome III consensus and here for the first time validated in a large group of NERD patients.

Moreover, if we look at the prevalence of typical reflux symptoms in our population, we found that NERD pH-POS with positive SAP and negative SAP patients more often experienced regurgitation episodes than did patients with FH, with an increasing frequency during the recording period, suggesting that they have a GORD symptom pattern more similar to that of patients with erosive oesophagitis and Barrett’s oesophagus than with FH.^1^,^2^,^3^,^4^ The percentages of patients reporting heartburn were similar in our subgroups, but patients with FH reported more frequently heartburn during the monitoring. This certainly could have been influenced by psychosocial factors as suggested by Watson *et al*.^11^ Unfortunately, in the current study, psychological profiles were not collected in all patients. While available data (results not shown) indicating more frequently anxiety and/or depression in the FH patient group are consistent with recently published data on functional dyspepsia by van Odenhove *et al*,^24^ we are deferring further conclusions to an adequately designed and powered study evaluating the contribution of psychology features in symptom generation in patients with functional oesophageal disorders.

Finally, some demographic features were different in our subgroups. We found that NERD pH-POS patients independently of the symptom association were more frequently male, had a higher mean body mass index and an increased prevalence of hiatal hernia, in agreement with those studies considering these demographic and clinical parameters as risk factors for the development of GORD and, particularly, abnormal pH monitoring.^10^,^25^,^26^,^27^ These features may help to identify patients with NERD and with an abnormal pH-impedance monitoring. Moreover, we demonstrated a higher prevalence of female gender in the FH subgroup, as it has been reported in the functional dyspepsia population and generally in patients with functional disorders.^17^,^28^,^29^,^30^ No differences based on age, tobacco use, alcohol and coffee consumption, prevalence of *H pylori* infection could be demonstrated in our population. This is in agreement with various studies^20^,^27^ showing that these factors are not peculiar of anyone of our subgroups.

One limitation of our study is the lack of outcome prospective data as no information on the response to a standardised therapeutic approach in the described patient groups are currently available. However, considering the previous response to PPI treatment referred to by the majority of our patients before entering the study we can argue that patients with abnormal distal oesophageal acid exposure and/or positive symptom association and dyspeptic symptoms do respond better to antisecretive treatment compared to patients with negative symptom association and dyspeptic symptoms. Evaluating patients on a Mediterranean diet could also be regarded as a shortcoming of the present study with the argument that it was not refluxogenic enough to induce symptoms. While being cognisant of this problem we chose the Mediterranean diet in order to be able to compare the results in patients with NERD with those collected in healthy Italian volunteers and, on the other hand, trying to minimise diet-induced variation in the amount of gastro-oesophageal reflux. Last, but not least, the 30 day washout period before the upper endoscopy in patients using acid suppressive medication could have been too short for lesions to develop. Still, in our experience, this represents the maximum we can ask of patients in order to obtain good compliance and without them dropping out from the study. Moreover, the incidence of recurring erosive lesions after a shorter period of PPI cessation remains unknown.

In conclusion, the present study underscores the important overlap between functional dyspepsia and NERD. This argues for the need to monitor dyspeptic symptoms and both acid and non-acid reflux episodes in patients primarily diagnosed with NERD to help identify the proportion of patients with true FH, who should, preferably, be included in the overall population with functional gastrointestinal disorders and should not be considered as a GORD subgroup any more. We believe this deliberate separation would spare these patients from wasteful and protracted courses of years of acid suppression and, above all, prevent potentially disastrous exposure to surgical options. Prospective outcome data are now needed to show the clinical significance of these findings in respect of future definition of NERD. Of particular interest would be the prospective evaluation of the response to acid suppressive therapy of NERD pH-POS and NERD pH-NEG/SAP+ for acid patients against NERD pH-NEG/SAP+ for non-acid and the response to reflux reducing therapies of NERD pH-POS and NERD pH-NEG/SAP+ for acid and non-acid patients against NERD pH-NEG/SAP− patients. This possible redefinition as suggested in the present manuscript might impact upon the epidemiology, pathophysiology and natural history of the respective disorders.
